# Dammarane-type saponins from steamed leaves of *Panax Notoginseng*

**DOI:** 10.1007/s13659-011-0036-2

**Published:** 2011-12-16

**Authors:** Qing Liu, Jun-Jiang Lv, Min Xu, Dong Wang, Hong-Tao Zhu, Chong-Ren Yang, Ying-Jun Zhang

**Affiliations:** 1 State Key Laboratory of Phytochemistry and Plant Resources in West China, Kunming Institute of Botany, Chinese Academy of Sciences, Kunming, 650201 China; 2 Graduate University of Chinese Academy of Sciences, Beijing, 100049 China; 3 Weihe Biotech Research and Development Center, Yuxi, 653101 China

**Keywords:** *Panax notoginseng*, steamed leave, dammarane-type saponin, notoginsenoside

## Abstract

Four new dammarane-type triterpenoid saponins, namely notoginsenosides SFt_1_-SFt_4_ (**1–4**) were isolated from the steamed leaves of *Panax notoginseng* (Burk.) F. H. Chen (Araliaceae), together with 17 known saponins. Their structures were established on the basis of detailed spectroscopic analyses and acidic hydrolysis. The known ginsenosides Rk2 and Rh3 were obtained from *P. notoginseng* for the first time. All of these new saponins showed no *in vitro* cytotoxicity against five human cancer cell lines (HL-60, SMMC-7712, A-549, MCF-7, and SW480).

**Electronic Supplementary Material:**

Supplementary material is available for this article at 10.1007/s13659-011-0036-2 and is accessible for authorized users.

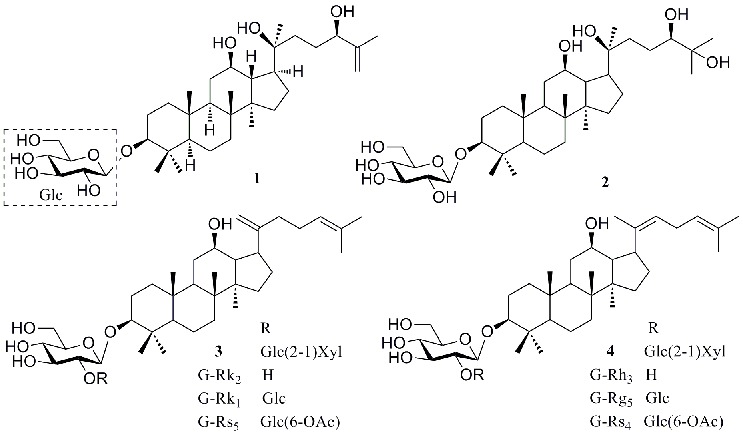

## Introduction

*Panax notoginseng* (Burk.) F. H. Chen (Araliaceae), a famous traditional Chinese medicinal (TCM) herb, is mainly cultivated in Yunnan and Guangxi province of China. Both roots and leaves have been used medicinally by the local people of its growing area for a long time.[1] Traditionally, the roots have been used in both raw and processed forms. The raw one has been mainly used for injuries from falls and removing blood stasis, while the processed one has been used as a tonic to promote blood circulation.[2] The leaves had the similar effects on hematological and cardiovascular systems as the roots. Dammarane-type triterpenoid saponins are known to be the main bioactive constituents in *P. notoginseng*. So far, more than 70 saponins have been isolated from different organs of this herbs.[3] Our previous study on the steamed roots of *P. notoginseng* resulted in the identification of five new and 23 known dammarane-type saponins, part of which are the minor constituents in non-processed roots.[4] As part of our continuing search for new saponins from *Panax* plants, a detailed chemical investigation on the steamed leaves of *P. notoginseng* was carried out, which led to the isolation of four new dammarane-type triterpenoid saponins (**1**–**4**), along with 17 known ones. Their structures were established based on the detailed spectroscopic analyses and acidic hydrolysis. The new saponins **1**–**4** were tested for their cytotoxic activities against five human cancer cell lines (HL-60, SMMC-7712, A-549, MCF-7, and SW480) *in vitro*.

*To whom correspondence should be addressed. E-mail: zhangyj@mail.kib.ac.cn

**Table 1. $^1$H NMR data (500 MHz, $C_5D_5N$) of compounds 1–4 ($\delta$ in ppm, $J$ in Hz).**

| No. | 1 | 2 | 3 [a] | 4 [a] |
|---|---|---|---|---|
| 1 | 1.50 m; 0.74 br. s | 1.48 m; 0.75 br. s | 1.78 m; 0.75 m | 1.74 m; 0.74 m |
| 2 | 1.90 m; 1.47 m | 2.20 m; 2.08 m | 2.18 br. d (11.6); 1.80 m | 2.18 dd (13.5, 3.0); 1.78 m |
| 3 | 3.37 dd (11.5, 4.0) | 3.38 dd (11.5, 3.5) | 3.29 dd (12.0, 4.0) | 3.28 dd (12.0, 4.4) |
| 5 | 0.72 t (11.5) | 0.73 t (11.5) | 0.68 t (11.2) | 0.67 t (11.2) |
| 6 | 1.52 m; 1.40 m | 1.48 m; 1.34 m | 1.40 m | 1.47 m; 1.04 m |
| 7 | 1.48 m; 1.20 br. d (11.5) | 1.43 m; 1.22 br. d (11.0) | 1.46 m; 1.38 m | 1.42 m; 1.20 br. d (10.5) |
| 9 | 1.42 m | 1.40 m | 1.38 m | 1.39 m |
| 11 | 2.17 m; 1.39 m | 2.00 m; 1.37 m | 2.05 m | 1.97 m; 1.35 m |
| 12 | 4.25 m | 4.25 m | 3.87 m | 3.91 m |
| 13 | 2.12 m | 2.06 m | 3.68 m | 3.68 m |
| 15 | 2.02 m; 1.52 m | 1.56 m; 1.02 m | 1.48 m; 1.10 m | 1.50 m; 1.04 m |
| 16 | 2.22 m; 1.81 m | 2.20 m; 2.08 m | 2.05 m; 1.56 br. s | 1.97 m; 1.48 m |
| 17 | 2.37 m | 2.37 m | 2.79 m | 2.77 m |
| 18 | 0.95 s | 0.94 s | 0.95 s | 0.94 s |
| 19 | 0.78 s | 0.79 s | 0.78s | 0.78 s |
| 21 | 1.44 s | 1.46 s | 5.14 s | 1.81 s |
| 22 | 2.04 m; 1.54 m | 1.90 m; 1.83 m | 2.46 m; 2.29 m | 5.49 t (6.8) |
| 23 | 2.09 m | 2.53 m; 1.80 m | 2.96 m; 2.37 m | 2.77 m |
| 24 | 4.41 m | 3.82 d (10.0) | 5.28 m | 5.21 t (7.2) |
| 26 | 1.88 s | 1.54 s | 1.64 s | 1.60 s |
| 27 | 5.26 s; 4.91 s | 1.51 s | 1.58 s | 1.56 s |
| 28 | 1.31 s | 1.32 s | 1.28 s | 1.27 s |
| 29 | 0.98 s | 1.00 s | 1.10 s | 1.09 s |
| 30 | 0.95 s | 0.96 s | 0.99 s | 0.99 s |
| 1' | 4.96 d (7.8) | 4.97 d (7.7) | 4.94 d (7.6) | 4.93 d (7.7) |
| 2' | 4.02 m | 4.06 t (8.5) | 4.09 m | 4.08 m |
| 3' | 4.01 m | 4.25 m | 4.37 m | 4.36 m |
| 4' | 3.93 m | 3.90 m | 4.13 m | 4.22 m |
| 5' | 4.25 m | 4.03 m | 4.28 m | 4.24 m |
| 6' | 4.60 dd (11.5, 5.0); 4.41 m | 4.61 br. d (11.5); 4.42 dd (11.5, 5.0) | 4.58 br. d (11.6); 3.97 m | 4.57 br. d (10.0); 4.34 m |
| 1'' | | | 5.53 d (7.6) | 5.52 d (7.7) |
| 2'' | | | 4.23 m | 4.20 m |
| 3'' | | | 4.34 m | 4.27 m |
| 4'' | | | 4.13 m | 4.11 m |
| 5'' | | | 4.29 m | 4.35 m |
| 6'' | | | 4.48 dd (11.0, 1.8); 4.27 m | 4.47 dd (11.2, 2.4); 4.26 m |
| 1''' | | | 5.42 d (6.5) | 5.41 d (6.5) |
| 2''' | | | 4.09 m | 4.12 m |
| 3''' | | | 4.36 m | 4.32 m |
| 4''' | | | 4.13 m | 4.14 m |
| 5''' | | | 3.97 m | 3.85 m |

[a] Recorded at 400 MHz.

## Results and Discussion

After defatted by petroleum ether, the ethanolic extract of steamed leaves of *P. notoginseng* was subjected to column chromatography repeatedly over Diaion 101 resin, silica gel, MCI gel CHP 20P, RP-8, and RP-18, and preparative-HPLC to afford four new dammarane-type triterpenoid saponins, namely, notoginsenosides $SFt_1$–$SFt_4$ (**1**–**4**), together with 17 known compounds.

The known compounds were identified as ginsenoside R10,[5] ginsenosides $Rk_2$,[6] $Rh_3$,[6] $Rk_1$,[6] $Rg_5$,[6] $Rs_5$[7] and $Rs_4$,[7] 20(R/S)-ginsenosides $Rh_2$,[8] $Rg_3$[9] and $Rs_3$,[10] 20(R/S)-notoginsenoside $Ft_1$,[11] ginsenosides $F_2$,[8] $Rb_3$,[8] Rc [8] and Fc,[12] gypenoside IX,[8] and 20(*R*/*S*)-protopanaxtriol,[13] respectively, by comparison the spectroscopic and physical data with those previously reported values.

Compound **1** was a white amorphous powder. The molecular formula $C_{36}H_{62}O_9$ was deduced from the HRESIMS data (*m/z* 673.4067, $[M + Cl]^-$). The $^1$H and $^{13}$C NMR spectroscopic data indicated the presence of a protopanaxdiol aglycone [$\delta_C$ 26.8 ($CH_2$, C-2), 88.8 (CH, C-3) and 18.5 ($CH_2$, C-6)][13] and a hexosyl unit [$\delta_H$ 4.96 (1H, d, $J$ = 7.8 Hz), $\delta_H$ 107.0 and 63.1]]. Acidic hydrolysis of **1** gave D-glucose as the sole sugar moiety, which was determined to have a $\beta$ configuration on the basis of the large coupling constants of the anomeric proton [$\delta_H$ 4.96 (1H, d, $J$ = 7.8 Hz)]. The aforementioned data demonstrated that compound **1** should be a protopanaxdiol triterpenoid saponin. Compared with protopanaxdiol,[14] the aglycone of **1** possesses a terminal double bonds [$\delta_H$ 5.26, 4.91 (each 1H, s), $\delta_H$ 109.9 ($CH_2$) and 150.0 (quaternary carbon)] and an oxygenatedmethine, instead of a methyl (C-27) and a methylene (C-24) in protopanaxdiol. The structure of **1** was further deduced by HMBC and HSQC experiments. The HMBC correlations (Figure 1) of $\delta_H$ 2.09 (m, $CH_2$) with $\delta_C$ 73.1 (C-20), $\delta_H$ 4.41 (m, oxygenated CH) and 1.88 (s, $CH_3$) with $\delta_C$ 109.9 and 150.0 (terminal double bond), the glucosyl H-1' ($\delta_H$ 4.96) with C-3 ($\delta_C$ 88.8) of aglycone were observed. The configuration of the C-24 was established to be *R* by the comparison of the $^{13}$C NMR data with those of the C-24 epimers of ginsenoside-

**Figure 1.** The key HMBC correlations (H→C) of compound **1**.

**Table 2.** $^{13}$**C NMR data (125 MHz,** $C_5D_5N$**) of compounds 1–4 (*δ* in ppm).**

| No. | 1 | 2 | 3[a] | 4[a] | No. | 1 | 2 | 3[a] | 4[a] |
|---|---|---|---|---|---|---|---|---|---|
| 1 | 39.2 ($CH_2$) | 39.7 ($CH_2$) | 39.8 ($CH_2$) | 39.3 ($CH_2$) | 25 | 150.0 (C) | 72.8 (C) | 131.2 (C) | 131.3 (C) |
| 2 | 26.8 ($CH_2$) | 26.8 ($CH_2$) | 26.8 ($CH_2$) | 28.8 ($CH_2$) | 26 | 18.5 ($CH_3$) | 26.2 ($CH_3$) | 25.8 ($CH_3$) | 25.8 ($CH_3$) |
| 3 | 88.8 (CH) | 88.8 (CH) | 88.9 (CH) | 88.9 (CH) | 27 | 109.9 ($CH_2$) | 26.0 ($CH_3$) | 17.8 ($CH_3$) | 17.8 ($CH_3$) |
| 4 | 39.7 (C) | 39.2 (C) | 39.3 (C) | 40.3 (C) | 28 | 28.2 ($CH_3$) | 28.2 ($CH_3$) | 28.1 ($CH_3$) | 28.1 ($CH_3$) |
| 5 | 56.4 (CH) | 56.4 (CH) | 56.4 (CH) | 56.4 (CH) | 29 | 15.9 ($CH_3$) | 15.8 ($CH_3$) | 16.7 ($CH_3$) | 15.8 ($CH_3$) |
| 6 | 18.5 ($CH_2$) | 18.5 ($CH_2$) | 18.5 ($CH_2$) | 18.5 ($CH_2$) | 30 | 17.1 ($CH_3$) | 17.1 ($CH_3$) | 17.0 ($CH_3$) | 17.1 ($CH_3$) |
| 7 | 35.2 ($CH_2$) | 35.2 ($CH_2$) | 35.2 ($CH_2$) | 35.4 ($CH_2$) | 1' | 107.0 (CH) | 107.0 (CH) | 104.8 (CH) | 104.9 (CH) |
| 8 | 40.0 (C) | 40.0 (C) | 40.2 (C) | 39.8 (C) | 2' | 75.8 (CH) | 75.8 (CH) | 83.0 (CH) | 83.0 (CH) |
| 9 | 50.4 (CH) | 50.5 (CH) | 48.2 (CH) | 50.9 (CH) | 3' | 78.8 (CH) | 78.8 (CH) | 78.7 (CH) | 78.7 (CH) |
| 10 | 37.0 (CH) | 37.0 (CH) | 37.0 (CH) | 37.1 (CH) | 4' | 71.0 (CH) | 71.0 (CH) | 71.1 (CH) | 71.2 (CH) |
| 11 | 32.3 ($CH_2$) | 32.1 ($CH_2$) | 32.6 ($CH_2$) | 32.3 ($CH_2$) | 5' | 78.4 (CH) | 78.4 (CH) | 78.3 (CH) | 78.4 (CH) |
| 12 | 71.9 (CH) | 71.9 (CH) | 72.4 (CH) | 72.6 (CH) | 6' | 63.1 ($CH_2$) | 63.1 ($CH_2$) | 63.0 ($CH_2$) | 63.0 ($CH_2$) |
| 13 | 48.6 (CH) | 48.6 (CH) | 52.8 (CH) | 50.8 (CH) | 1'' | | | 103.2 (CH) | 103.2 (CH) |
| 14 | 51.8 (C) | 51.8 (C) | 51.2 (C) | 50.5 (C) | 2'' | | | 84.6 (CH) | 84.6 (CH) |
| 15 | 32.3 ($CH_2$) | 31.5 ($CH_2$) | 32.6 ($CH_2$) | 32.7 ($CH_2$) | 3'' | | | 78.0 (CH) | 78.0 (CH) |
| 16 | 26.9 ($CH_2$) | 26.8 ($CH_2$) | 30.9 ($CH_2$) | 26.8 ($CH_2$) | 4'' | | | 71.7 (CH) | 71.8 (CH) |
| 17 | 54.9 (CH) | 54.6 (CH) | 50.8 (CH) | 51.0 (CH) | 5'' | | | 77.9 (CH) | 77.9 (CH) |
| 18 | 16.4 ($CH_3$) | 16.4 ($CH_3$) | 16.5 ($CH_3$) | 16.5 ($CH_3$) | 6'' | | | 62.8 ($CH_2$) | 62.8 ($CH_2$) |
| 19 | 16.8 ($CH_3$) | 16.8 ($CH_3$) | 15.8 ($CH_3$) | 16.7 ($CH_3$) | 1''' | | | 106.5 (CH) | 106.5 (CH) |
| 20 | 73.1 (C) | 73.3 (C) | 155.5 (C) | 140.2 (C) | 2''' | | | 76.0 (CH) | 76.0 (CH) |
| 21 | 27.5 ($CH_3$) | 27.5 ($CH_3$) | 108.0 ($CH_2$) | 13.2 ($CH_3$) | 3''' | | | 77.8 (CH) | 77.9 (CH) |
| 22 | 31.4 ($CH_2$) | 27.0 ($CH_2$) | 33.8 ($CH_2$) | 123.6 (CH) | 4''' | | | 70.7 (CH) | 70.8 (CH) |
| 23 | 30.7 ($CH_2$) | 33.9 ($CH_2$) | 27.1 ($CH_2$) | 27.5 ($CH_2$) | 5''' | | | 67.5 ($CH_2$) | 67.5 ($CH_2$) |
| 24 | 76.1 (CH) | 80.1 (CH) | 125.4 (CH) | 123.8 (CH) | | | | | |

[a]Recorded at 100 MHz.

Rg7[15] and majoroside-F2.[16] The lower-field shifted $^{13}$C NMR data of C-24 ($\delta_C$ 76.1) and C-26 ($\delta_C$ 109.9) [majoroside-$F_2$: $\delta_C$ 75.8 (C-24*S*), 109.3 (C-26); ginsenoside-$Rg_7$: $\delta_C$ 76.2 (C-24*R*)), 110.2 (C-26)] clearly confirmed the configuration of the C-24 hydroxyl group in **1**. Accordingly, the structure of **1** was elucidated and named as notoginsenoside $SFt_1$.

Compound **2**, a white amorphous powder, had a molecular formula $C_{36}H_{64}O_{10}$ on the basis of the HRESIMS (*m/z* 691.4150, $[M + Cl]^-$). The $^1$H and $^{13}$C NMR spectroscopic data resembled that of **1**, except for the loss of the double bond and the appearance of an additional oxygen-bearing quaternary carbon signal at $\delta_C$ 72.8. In the HMBC spectrum, the correlations of $\delta_H$ 1.54, 1.51 (each 3H, s, Me-26 and Me-27) and 3.82 (1H, d, $J$ = 10.0 Hz, oxygenated CH) with $\delta_C$ 72.8 (C-25), and $\delta_H$ 3.82 (1H, d, $J$ = 10.0 Hz, oxygenated CH) with $\delta_C$ 33.9 (C-23) and 27.0 (C-22) were observed, which confirmed that the additional hydroxyl group should be linking to C-25. The configuration of the C-24 (C-24: $\delta_C$ 80.1) was indicated to be 24*R* on the comparison with the chemical shift values of cyclocantogenin [$\delta_C$ 76.99 (C-24*S*)][17] and tarecilioside A [$\delta_C$ 80.5 (C-24*R*)].[18] Hence, the structure of compound **2** was established and named notoginsenoside $SFt_2$.

Compound **3** had the molecular formula $C_{47}H_{78}O_{16}$ based on the HRESI data (*m/z* 897.5215, $[M - H]^-$). The $^1$H and $^{13}$C NMR data revealed that **3** was similar to the known dammarane saponin, ginsenoside $Rk_1$,[6] except for an additional pentosyl unit. Acidic hydrolysis of **3** gave D-glucose and D-xylose as sugar residues. The large coupling constants of the anomeric protons ($J$ = 7.6, 7.6, 6.5 Hz) indicated the $\beta$ configurations of two glucosyl and one xylosyl moieties. Locations of the sugar moieties were established by the HMBC correlations of the inner glucosyl H-1' ($\delta_H$ 4.94), the middle glucosyl H-1'' ($\delta_C$ 5.53), and the xylosyl H-1''' ($\delta_H$ 5.42) with C-3 ($\delta_C$ 88.9), the inner glucosyl C-2' ($\delta_C$ 83.0) and the middle glucosyl C-2'' ($\delta_C$ 84.6), respectively. Accordingly, the structure of compound **3** was established and named as notoginsenoside $SFt_3$.

The molecular formula of compound **4** was deduced as $C_{47}H_{78}O_{16}$ by the HRESIMS (*m/z* 897.5235, $[M - H]^-$). The $^1$H and $^{13}$C NMR features of **4** were closely related to those of **3**, including the same trisaccharide moiety. The only difference was that the terminal double bonds between C-20 and C-21 in **3** were changed to be a middle double bond between C-20 and C-22 in **4**. This could be determined by comparison of the $^{13}$C NMR data with those of the known compound ginsenoside $Rg_5$ and further confirmed by HMBC correlations of $\delta_H$ 1.81 (Me, C-21) with $\delta_C$ 51.0 (C-17), 140.2 (C-20) and 123.6 (C-22). Therefore, the structure of compound **5** was deduced and named as notoginsenoside $SFt_4$.

Compounds **1**–**4** were tested for their cytotoxic activity against five human cancer cell lines, HL-60 myeloid leukemia, SMMC-7721 hepatocellular carcinoma, A-549 lung cancer, MCF-7 breast cancer, and SW480 colon cancer, applying the MTT method. However, none of them exhibited cytotoxicity at a concentration of 40 $\mu$M.

The present study indicated that the chemical constituents of the steamed leaves of *P. notoginseng* were significantly different from the un-processed ones.[8] After steamed, a mass of low-pole triterpenoid saponins came forth. Being similar with the roots, the processed and the air-dried leaves must have different pharmacological activities and quality standard.

## Experimental Section

**General Experimental Procedures**. Optical rotations were performed on a P-1020 polarimeter (JASCO, Tokyo, Japan). IR spectra were measured on a Bruker Tensor 27 spectrometer with KBr pellets. 1D and 2D NMR spectra were run on Bruker AM-400 and DRX-500 instruments operating at 400 and 500 MHz for $^1$H, and 100 and 125 MHz for $^{13}$C, respectively. Coupling constants are expressed in Hertz and chemical shifts are given on a ppm scale with tetramethylsilane as internal standard. The MS data were recorded on a VG Auto Spec-3000 spectrometer (VG, Manchester, U.K.) with glycerol as the matrix. HRESIMS were recorded on an API Qstar Pulsa LC/TOF spectrometer. GC analysis was run on a Shimadzu GC-14C gas chromatograph.

Column chromatography was performed with Diaion 101 resin (Tianjin Haiguang Chemical Co., Ltd. Tianjin, China), silica gel (200–300 mesh, Qingdao Makall Group Co., Ltd. Qingdao, China), MCI gel CHP 20P (Mitsubishi Chemical Co. Tokyo, Japan), Rp-8 gel, Rp-18 gel (40–60 $\mu$m, Merck, Darmstadt, Germany) and a 250 × 9.4 mm, i.d., 5$\mu$m Zorbax SB-$C_{18}$ column (Agilent, California, USA). Thin-layer chromatography (TLC) was carried out on silica gel H-precoated plates (Qingdao Makall Group Co., Ltd.) with $CHCl_3$/MeOH/$H_2O$ (8.5:1.5:0.1, 8:2:0.2 or 7:3:0.5, v/v), RP-8 and RP-18 precoated plates (Merck) with MeOH/$H_2O$ (7:3 or 8:2, v/v). Spots were detected by spraying with 10% $H_2SO_4$ in EtOH followed by heating.

**Plant Material.** Air-dried leaves of *P. notoginseng* were collected from Wenshan County, Yunnan province, China, in May, 2008. The raw leaves were mixed with appropriate amount of water and steamed at 120°C for 12 h, to give steamed leaves.

**Extraction and Isolation.** The steamed leaves of *P. notoginseng* (10 kg) were extracted with ethanol for three times (1.5 h × 3) under reflux. The ethanolic extract was concentrated under reduced pressure to small volume, and then defatted with petroleum ether for 5 times. After removal of the organic solvent, the defatted ethanolic extract was subjected to a Diaion 101 resin column, eluting with $H_2O$ and EtOH, successively. The EtOH elutes were combined and concentratedunder reduced pressure to give the total saponin fraction (850 g), 200 g of which was subjected to silica gel column chromatography (CC) (55 × 6.2 cm) eluted with $CHCl_3$/MeOH (95:5, v/v) and $CHCl_3$/MeOH/$H_2O$ (9:1:0.1 to 5:5:1, v/v) to afford 6 fractions (Fr. 1–6). Fr. 1 (12 g) was chromatographed over silica gel ($CHCl_3$/MeOH, 95:5 and 90:10, v/v), Rp-8 gel (MeOH/$H_2O$, 6:4-9:1, v/v), and MCI gel CHP 20P (MeOH/$H_2O$, 7:3–1:0, v/v) to afford 20(*R*/*S*)-protopanaxtriol (138 mg). Fr. 2 (25 g) was subjected to column chromatography on silica gel ($CHCl_3$/MeOH/$H_2O$, 95:5:0, 9:1:0.1, 8.5:1.5:0.1 and 8:2:0.2, v/v), Rp-8 gel (MeOH/$H_2O$, 6:4–9:1, v/v), and pre-HPLC (MeOH/$H_2O$, 89:11, v/v) to give ginsenoside R10 (12 mg), $Rk_2$ (11 mg), $Rh_3$ (7 mg) and $Rh_2$ (1.1 g). In a similar way, compounds **1** (140 mg), **2** (11 mg), **3** (9 mg), **4** (15 mg), $Rk_1$ (4 mg), $Rg_5$ (9 mg), $Rs_5$ (8 mg), $Rs_4$ (6 mg), $Rg_3$ (206 mg), $Rs_3$ (42 mg), $Ft_1$ (51 mg) and $F_2$ (8 mg) were obtained from Fr. 3 (31 g) and Fr. 4 (11 g), respectively. Each 1 gram sample of Fr. 5 (41 g) and Fr. 6 (36 g), respectively, was separated by Chromatorex ODS ($H_2O$/MeOH 1:1, v/v), silica gel ($CHCl_3$/MeOH/$H_2O$ 8:2:0.2–7:3:0.5) and Rp-8 gel (MeOH/$H_2O$, 6:4–9:1, v/v) to obtain the known compounds, gypenoside IX (450 mg), and ginsenosides $Rb_3$ (52 mg), Rc (48 mg) and Fc (158 mg).

**Notoginsenoside SFt₁ (1):** white amorphous powder; $[\alpha]_D^{24}$ +9.4 (*c* 0.20, MeOH); IR (KBr) $\nu_{max}$ 3442, 2947, 1640, 1078, 1022 $cm^{-1}$; $^1H$ and $^{13}C$ NMR data see Tables 1 and 2; FABMS *m/z* 637 $[M - H]^-$, 475 $[M - 162 (glucosyl) - H]^-$; HRESIMS *m/z* 673.4067 $[M + Cl]^-$ (calcd. for $C_{36}H_{62}O_9Cl$, 673.4082).

**Notoginsenoside $SFt_2$ (2):** white amorphous powder; $[\alpha]_D^{24}$ −3.1 (*c* 0.12, MeOH); IR (KBr) $\nu_{max}$ 3425, 2946, 1640, 1078, 1021 $cm^{-1}$; $^1H$ and $^{13}C$ NMR data see Tables 1 and 2; FABMS *m/z* 656 $[M - H]^-$, 493 $[M - 162 (glucosyl) - H]^-$; HRESIMS *m/z* 691.4150 $[M + Cl]^-$ (calcd. for $C_{36}H_{64}O_{10}Cl$, 691.4188).

**Notoginsenoside $SFt_3$ (3):** white amorphous powder; $[\alpha]_D^{24}$ −3.6 (*c* 0.28, MeOH); IR (KBr) $\nu_{max}$ 3424, 2943, 1639, 1076, 1044 $cm^{-1}$; $^1H$ and $^{13}C$ NMR data see Tables 1 and 2; FABMS *m/z* 897 $[M - H]^-$, 765 $[M - 132 (xyl) - H]^-$, 603 $[M - 132 (xyl) - 162 (glucosyl) - H]^-$; HRESIMS *m/z* 897.5215 $[M - H]^-$ (calcd. for $C_{47}H_{77}O_{16}$, 897.5211).

**Notoginsenoside $SFt_4$ (4):** white amorphous powder; $[\alpha]_D^{24}$ −7.2 (*c* 0.12, MeOH); IR (KBr) $\nu_{max}$ 3443, 2927, 1640, 1076, 1044 $cm^{-1}$; $^1H$ and $^{13}C$ NMR data see Tables 1 and 2; FABMS *m/z* 897 $[M - H]^-$, 765 $[M - 132 (xyl) - H]^-$, 603 $[M - 132 (xyl) - 162 (glucosyl) - H]^-$; HRESIMS *m/z* 897.5235 $[M - H]^-$ (calcd. for $C_{47}H_{77}O_{16}$, 897.5211).

**Acidic Hydrolysis of Compounds 1–4.** Compounds **1–4** (each 3–5 mg) were hydrolyzed with 2 M HCl/dioxane (1:1, 4 mL) under reflux for 6 h, respectively. The reaction mixture was extracted with $CHCl_3$ for five times (2 mL × 5). The aqueous layer was neutralized with 2 M NaOH and then dried to give a monosaccharide mixture. After that, a solution of the sugar mixture in pyridine (2 mL) was added to L-cysteine methyl ester hydrochloride (about 1.5 mg) and kept at 60°C for 1 h. Then trimethylsilylimidazole (about 1.5 mL) was added to the reaction mixture in ice-water base and kept at 60°C for 30 min. The mixture was subjected to GC analysis, run on a Shimadzu GC-14C gas chromatograph equipped with a 30 m × 0.32 mm i.d. 30QC2/AC-5 quartz capillary column and an $H_2$ flame ionization detector with the following conditions: column temperature, 180–280°C; programmed increase, 3 °C/min; carrier gas, $N_2$ (1 mL/min); injector and detector temperature, 250°C; injection volume, 4 $\mu$L; and split ratio, 1/50. The configuration of the sugar moiety was determined by comparing the retention time with the derivatives of the authentic samples.

**Cytotoxic Bioassay**. Human myeloid leukemia HL-60, hepatocellular carcinoma SMMC-7721, lung cancer A-549, breast cancer MCF-7, and colon cancer SW480 cell lines were used in the cytotoxic assay. The assay was performed by means of MTT [3-(4,5-dimethylthiazol-2-yl)-2,5-diphenlytetrezolium bromide] (Sigma, St. Louis, USA) method[19] in 96-well microplates. All the cells were cultured in RMPI1640 or DMEM medium (Hyclone, USA), supplemented with 10% fetal bovine serum (Hyclone, USA) in 5% $CO_2$ at 37 °C. 100 $\mu$L suspension was added to each well to seed cells in 96-well microplates, in which the tested samples were added with varied concentrations. After 48 hours incubation, MTT solution [5 mg/mL in phosphate buffered saline (PBS)] was added (20 $\mu$L/well), and the incubation continued for another 4h to give the formazan product. In each well 100 $\mu$L 20%

SDS was added after 100 $\mu$L medium was removed, and it was then incubated over night to make the formazan product dissolve completely. The absorbance of the solution was measured at 595 nm in Bio-Rad 680. Concentration of a compound inhibiting 50% of cell growth ($IC_{50}$) was calculated by the Reed and Muench method.

## Electronic Supplementary Material

Supplementary material is available in the online version of this article at http://dx.doi.org/10.1007/s13659-011-0036-2 and is accessible for authorized users.

## Acknowledgments

The authors are grateful to the members of the analytical group at State Key Laboratory of Phytochemistry and Plant Resources in West China, Kunming Institute of Botany, Chinese Academy of Sciences, for measuring all the spectroscopic data. This work was supported by the 973 Program of Ministry of Science and Technology of China (2011CB915503).

**Open Access** This article is distributed under the terms of the Creative Commons Attribution License which permits any use, distribution, and reproduction in any medium, provided the original author(s) and source are credited.

## Electronic supplementary material

Supplementary material, approximately 4.43 MB.
