# Supplementary material for: Dammarane-type saponins from steamed leaves of Panax Notoginseng
Source: Nat Prod Bioprospect. 2011 Dec 16;1(3):124–8. doi: 10.1007/s13659-011-0036-2 (PMC4131641; doi:10.1007/s13659-011-0036-2)

Electronic Supplementary Material

Dammarane-type saponins from steamed leaves of *Panax Notoginseng*

Qing LIU,[a] Jun-Jiang LV,[a,b] Min XU,[a] Dong WANG,[a] Hong-Tao ZHU,[a] Chong-Ren YANG,[a,c] and Ying-Jun ZHANG[a,*]

[a]State Key Laboratory of Phytochemistry and Plant Resources in West China, Kunming Institute of Botany, Chinese Academy of Sciences, Kunming 650201, China

[b]Guangxi Botanical Garden of Medicinal Plants, 189 Changgang Road, Nanning 530023, China

[c]Weihe Biotech Research and Development Center, Yuxi 653101, China

Received 22 November 2011; Accepted 11 December 2011

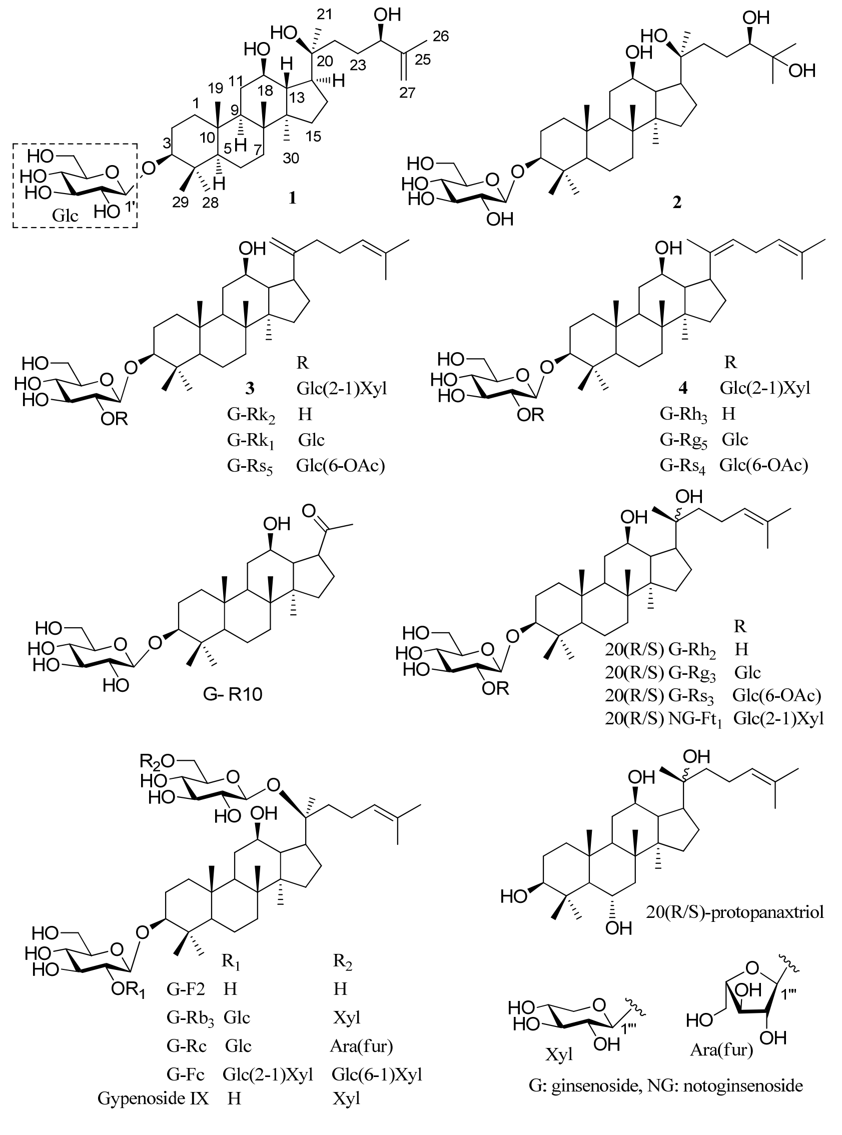

Structures of compounds

*To whom correspondence should be addressed. E-mail: zhangyj@mail.kib.ac.cn

S1 Supplementary data contents page

S2 ^{1}H NMR spectrum of notoginsenosides SFt_1 (**1**) in C_5D_5N

S3 ^{13}C NMR and DEPT spectra for notoginsenosides SFt_1 (**1**) in C_5D_5N

S4 HMQC spectrum of notoginsenosides SFt_1 (**1**) in C_5D_5N

S5 ^{1}H-^{1}H COSY spectrum of notoginsenosides SFt_1 (**1**) in C_5D_5N

S6 HMBC spectrum for notoginsenosides SFt_1 (**1**) in C_5D_5N

S7 ^{1}H NMR spectrum of notoginsenosides SFt_2 (**2**) in C_5D_5N

S8 ^{13}C NMR and DEPT spectra for notoginsenosides SFt_2 (**2**) in C_5D_5N

S9 HMQC spectrum of notoginsenosides SFt_2 (**2**) in C_5D_5N

S10 ^{1}H-^{1}H COSY spectrum of notoginsenosides SFt_2 (**2**) in C_5D_5N

S11 HMBC spectrum for notoginsenosides SFt_2 (**2**) in C_5D_5N

S12 ^{1}H NMR spectrum of notoginsenosides SFt_3 (**3**) in C_5D_5N

S13 ^{13}C NMR spectrum for notoginsenosides SFt_3 (**3**) in C_5D_5N

S14 ^{1}H-^{1}H COSY spectrum of notoginsenosides SFt_3 (**3**) in C_5D_5N

S15 HMBC spectrum for notoginsenosides SFt_3 (**3**) in C_5D_5N

S16 ^{1}H NMR spectrum of notoginsenosides SFt_4 (**4**) in C_5D_5N

S17 ^{13}C NMR spectrum for notoginsenosides SFt_4 (**4**) in C_5D_5N

S18 ^{1}H-^{1}H COSY spectrum of notoginsenosides SFt_4 (**4**) in C_5D_5N

S19 HMBC spectrum for notoginsenosides SFt_4 (**4**) in C_5D_5N

S2 ^{1}H NMR spectrum of notoginsenosides SFt_1 (1) in C_5D_5N

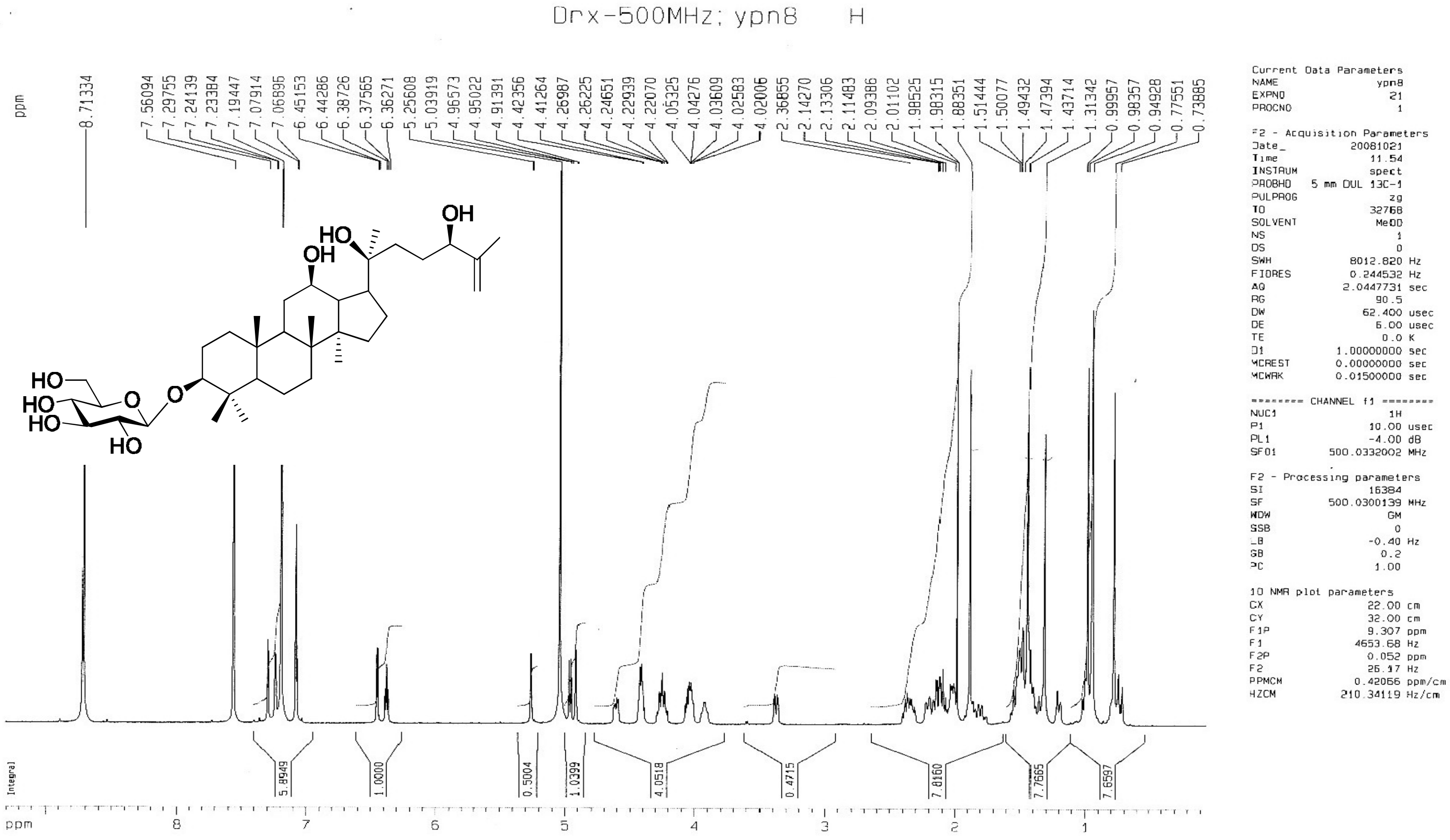

S3 ^{13}C NMR and DEPT spectra for notoginsenosides SFt$_1$ (1) in C_5D_5N

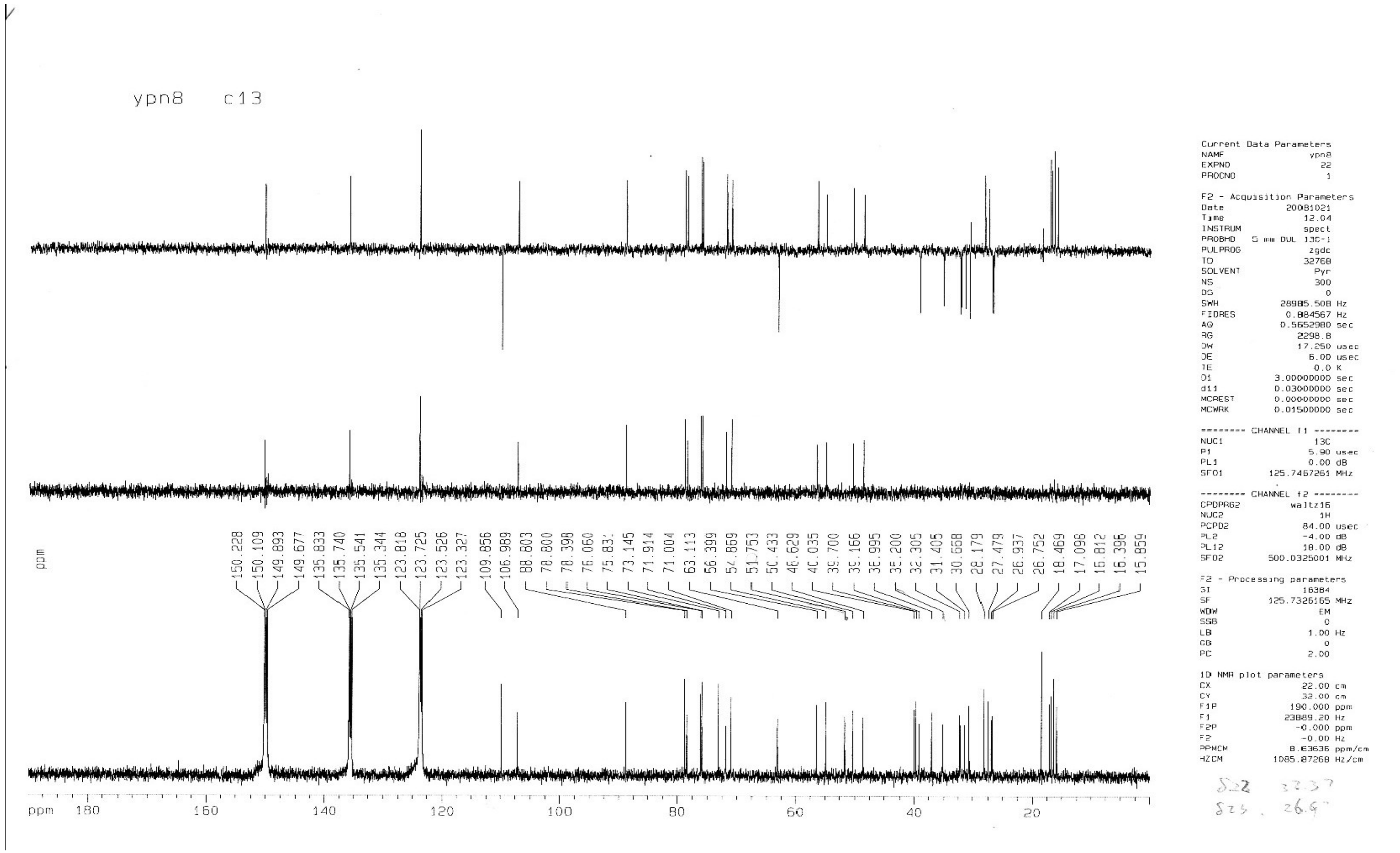

S4 HMQC spectrum of notoginsenosides SFt$_1$ (1) in C_5D_5N

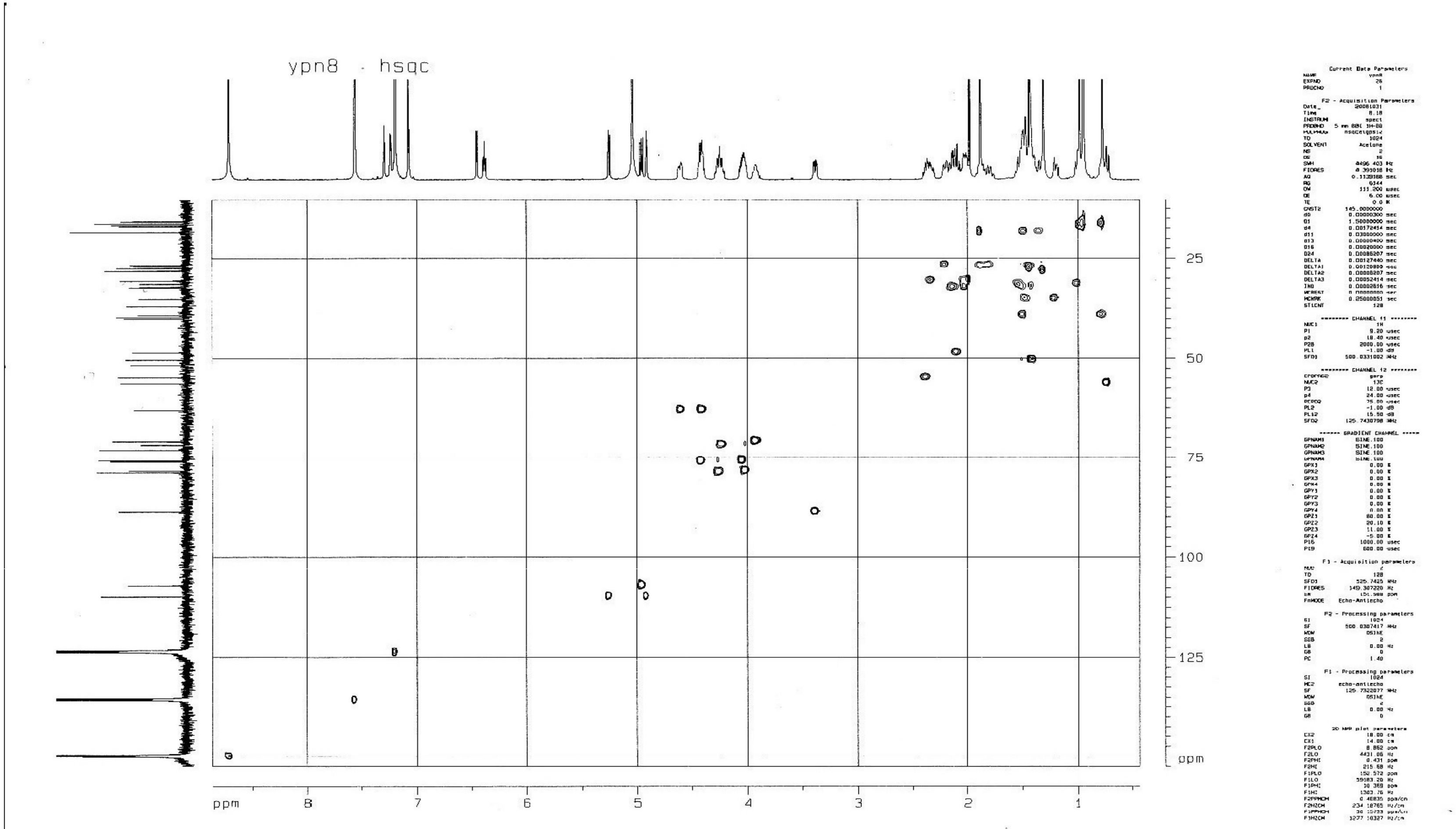

S5 ^{1}H-^{1}H COSY spectrum of notoginsenosides SFt$_1$ (1) in C_5D_5N

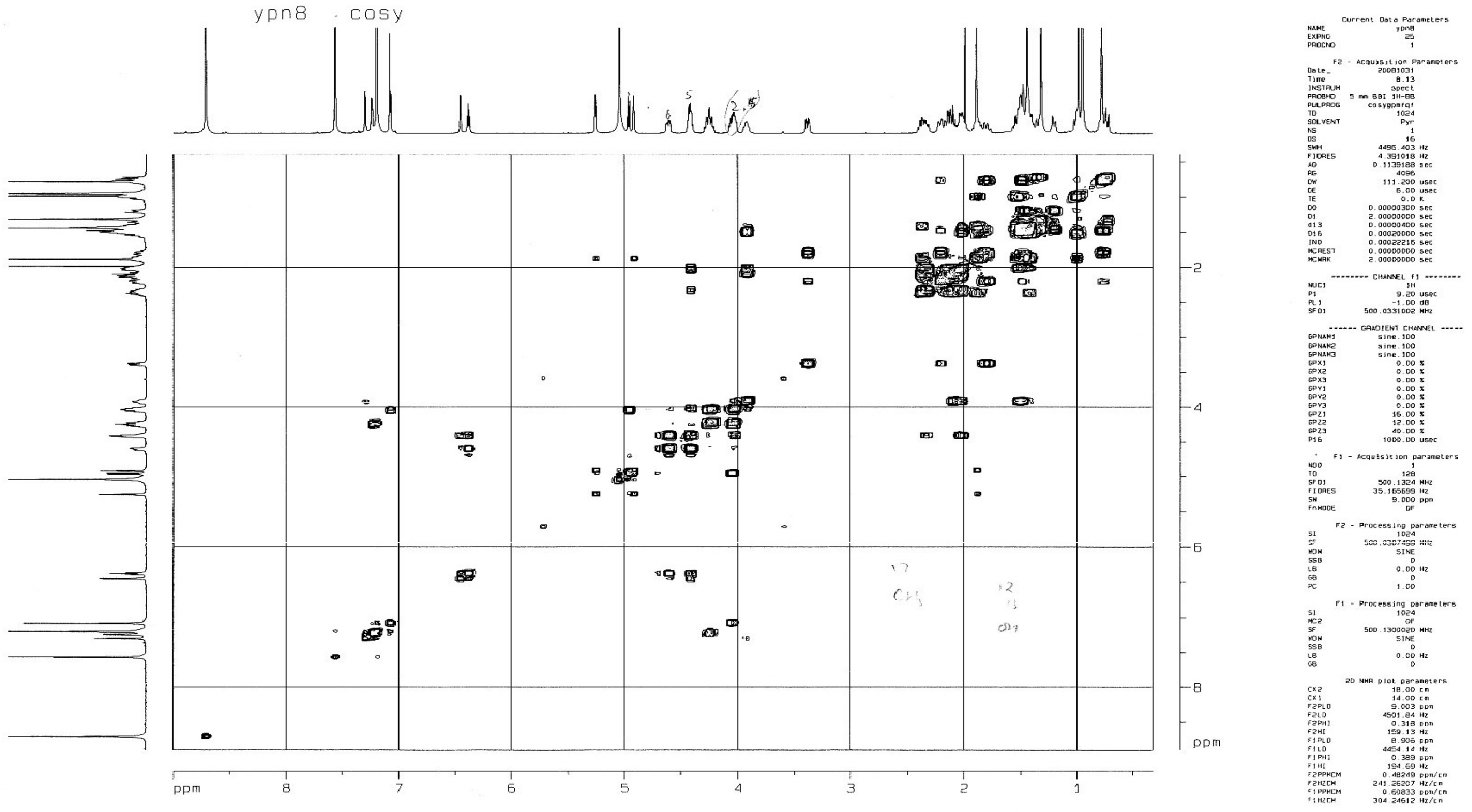

S6 HMBC spectrum for notoginsenosides SFt$_1$ (1) in C_5D_5N

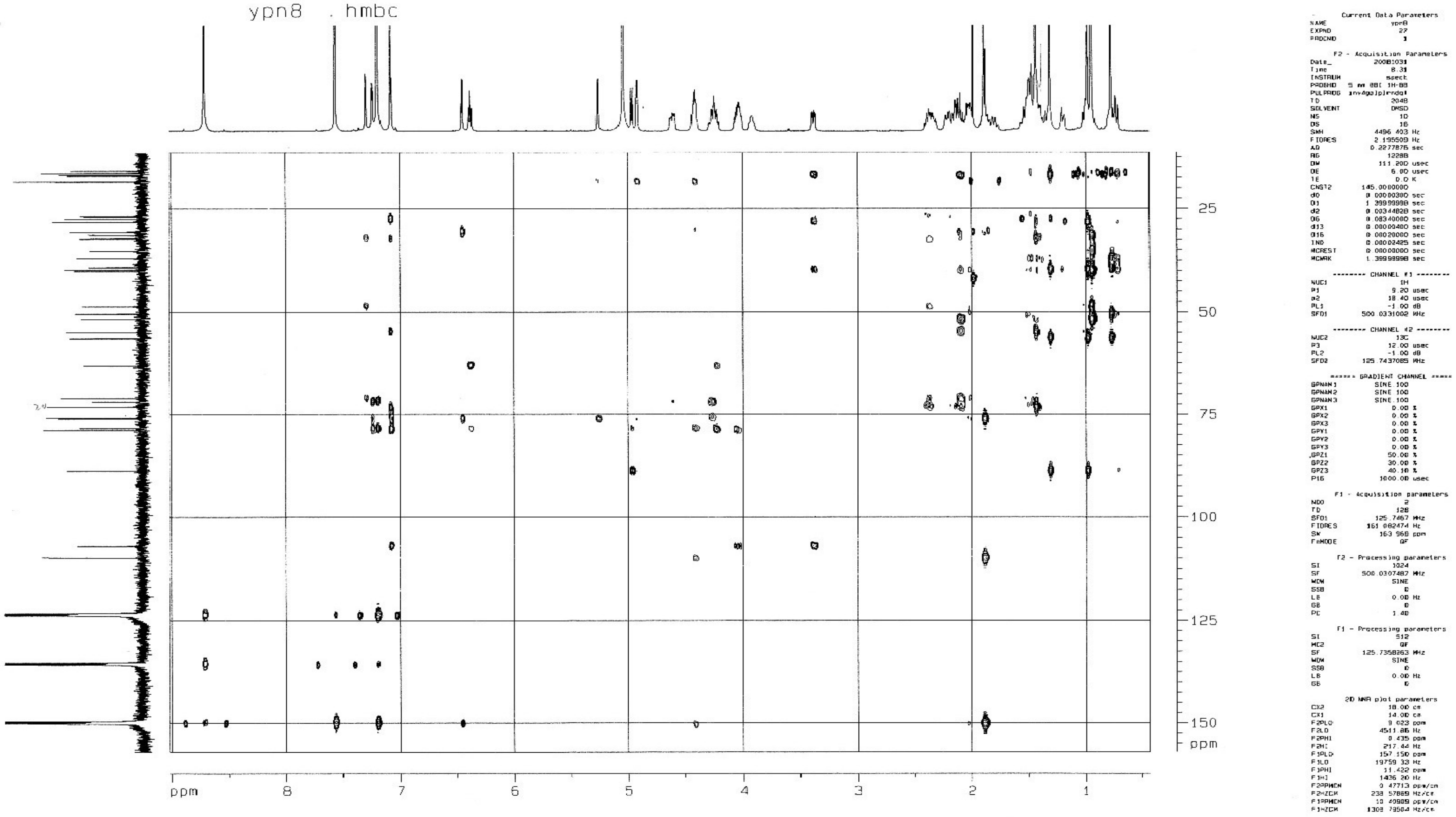

S7 ^{1}H NMR spectrum of notoginsenosides SFt$_2$ (2) in C_5D_5N

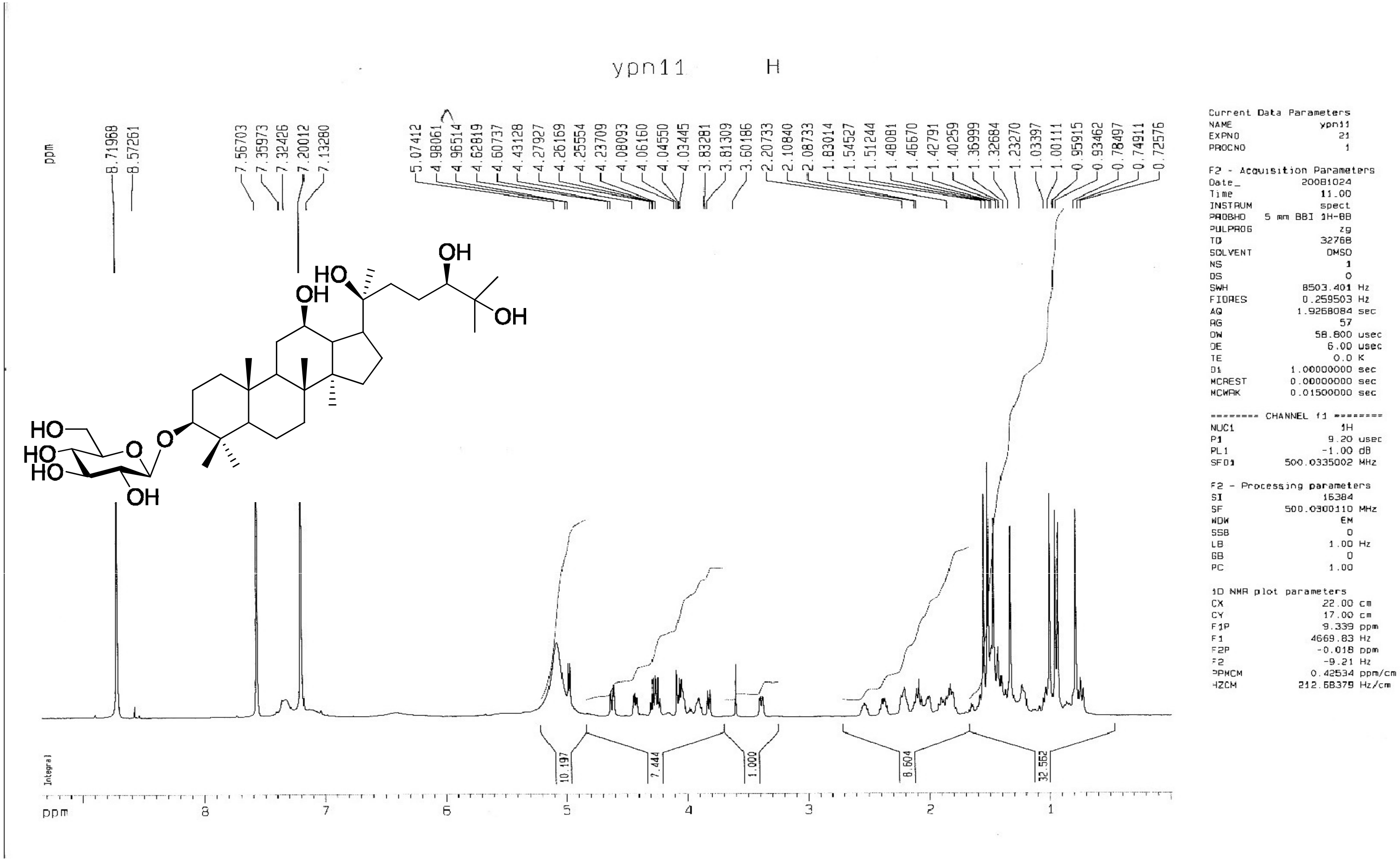

S8 ^{13}C NMR and DEPT spectra for notoginsenosides SFt$_2$ (2) in C_5D_5N

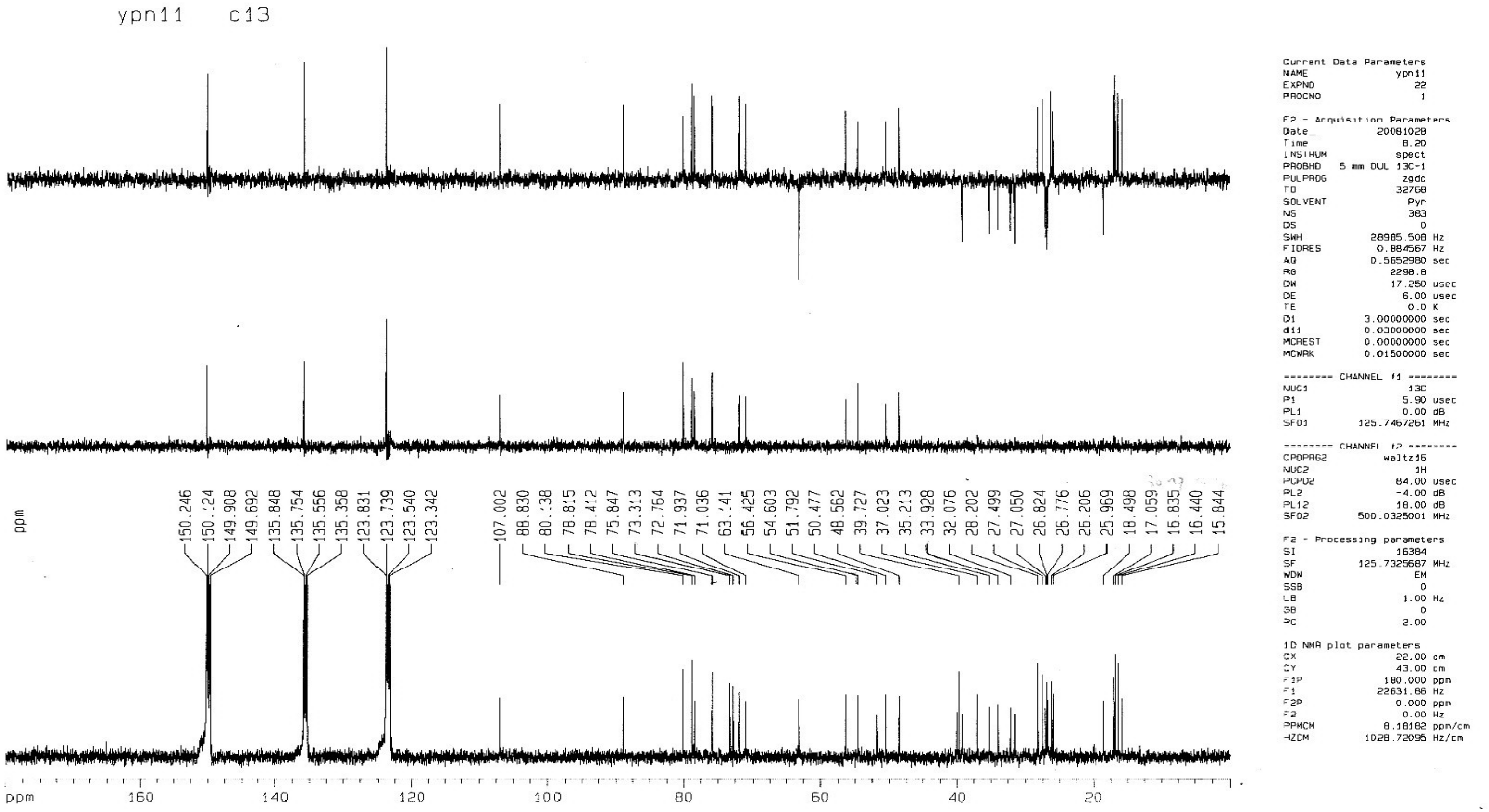

S9 HMQC spectrum of notoginsenosides SFt$_2$ (2) in C_5D_5N

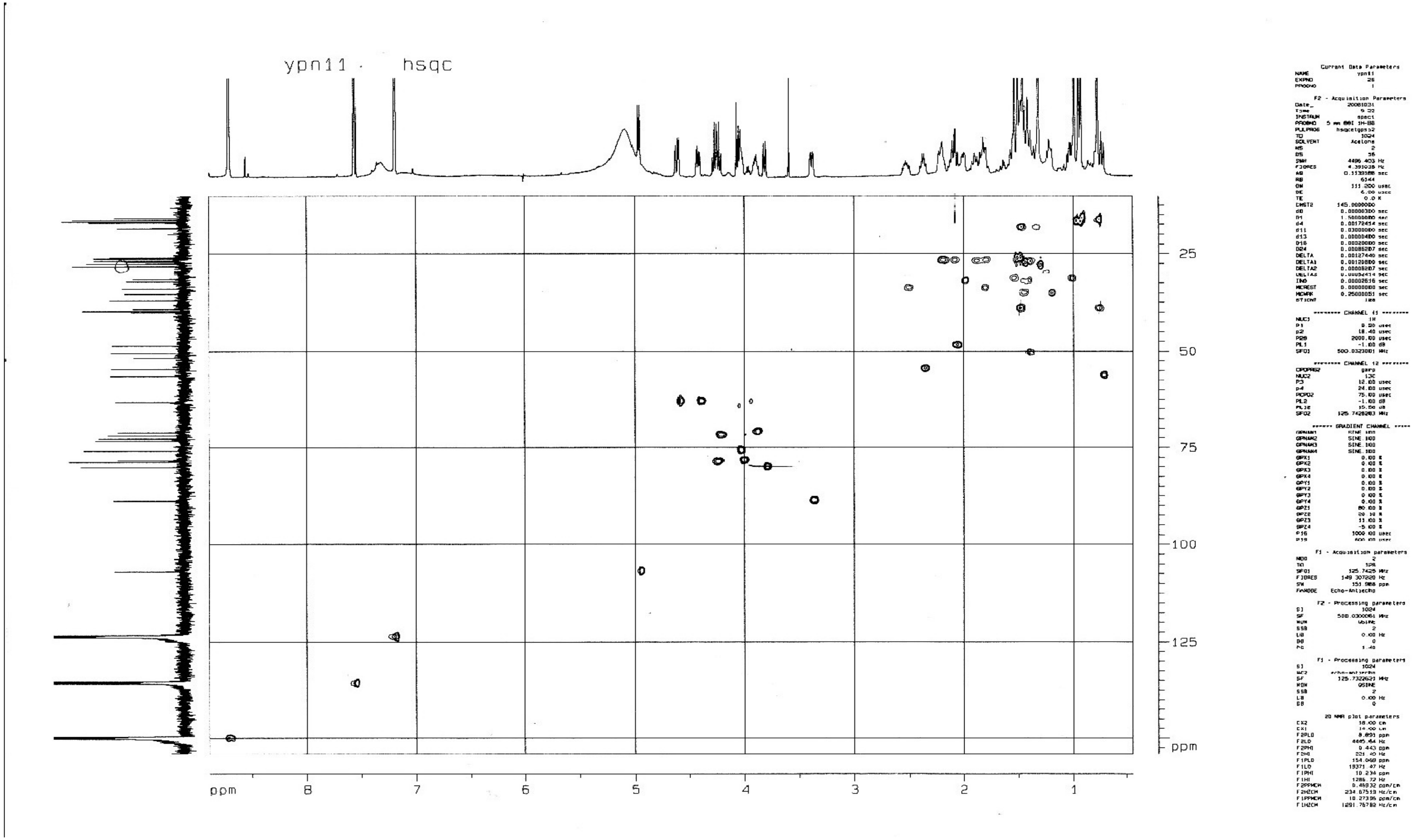

S10 ^{1}H-^{1}H COSY spectrum of notoginsenosides SFt_2 (2) in C_5D_5N

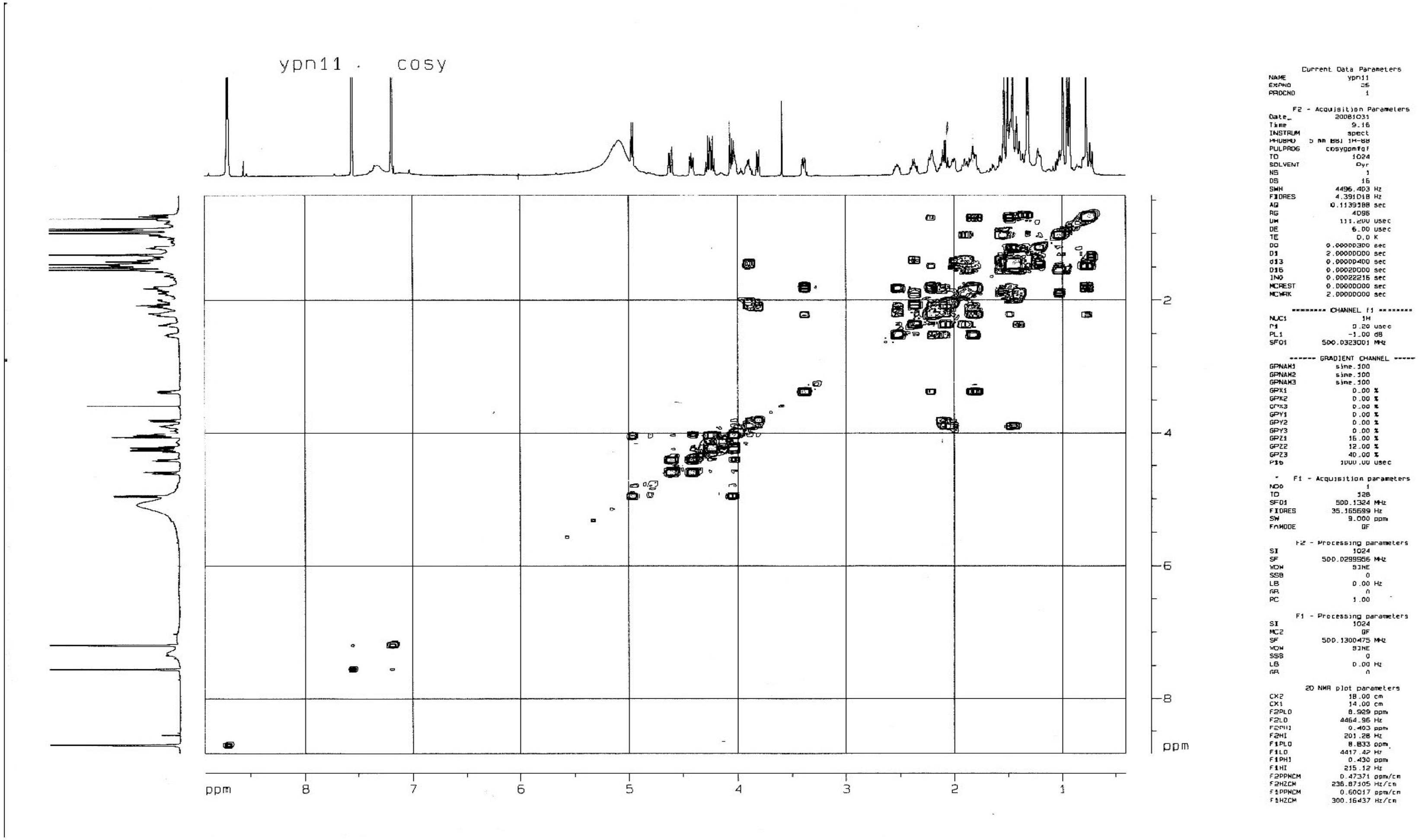

S11 HMBC spectrum for notoginsenosides SFt_2 (2) in C_5D_5N

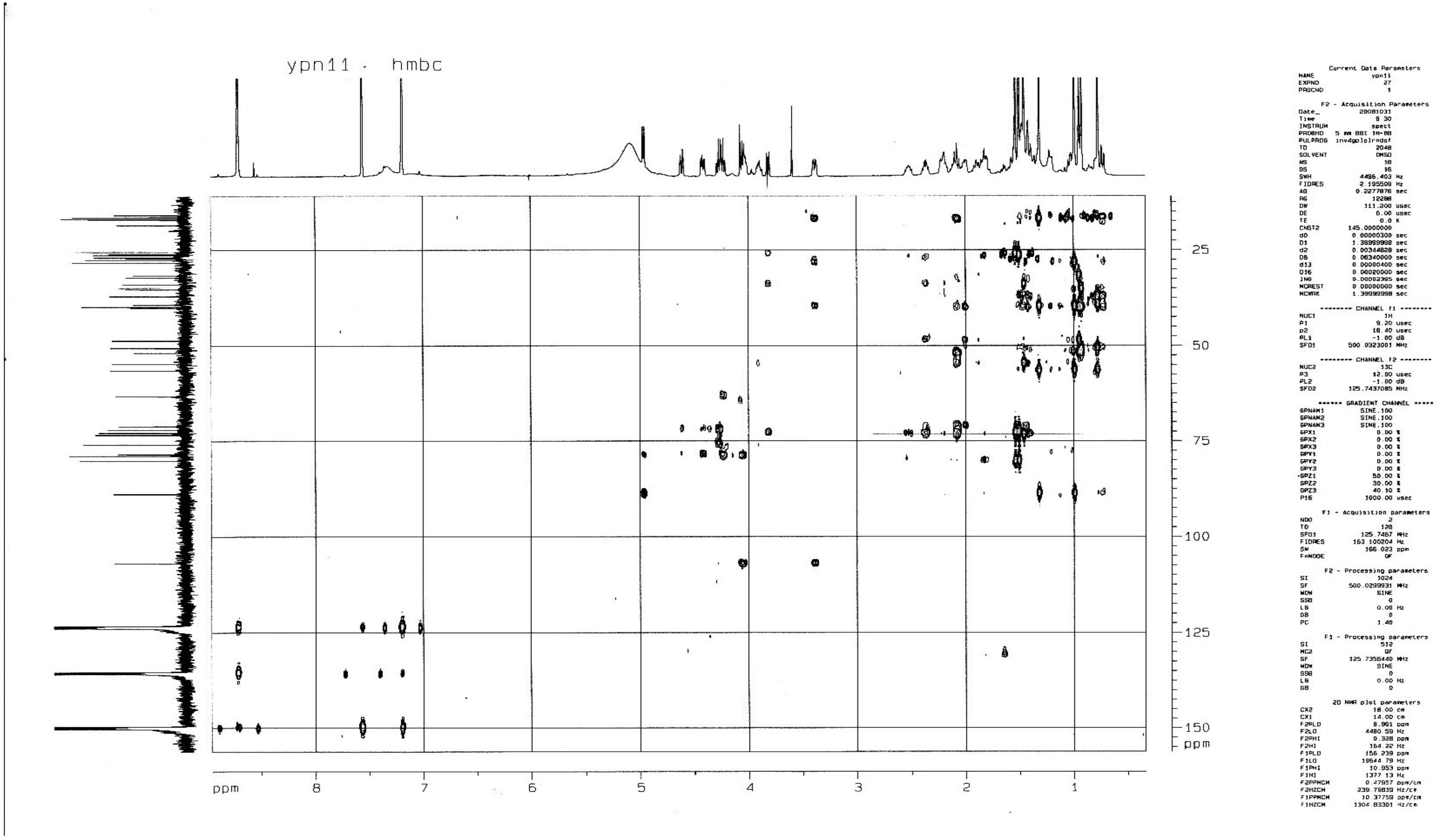

S12 ^{1}H NMR spectrum of notoginsenosides SFt$_3$ (3) in C_5D_5N

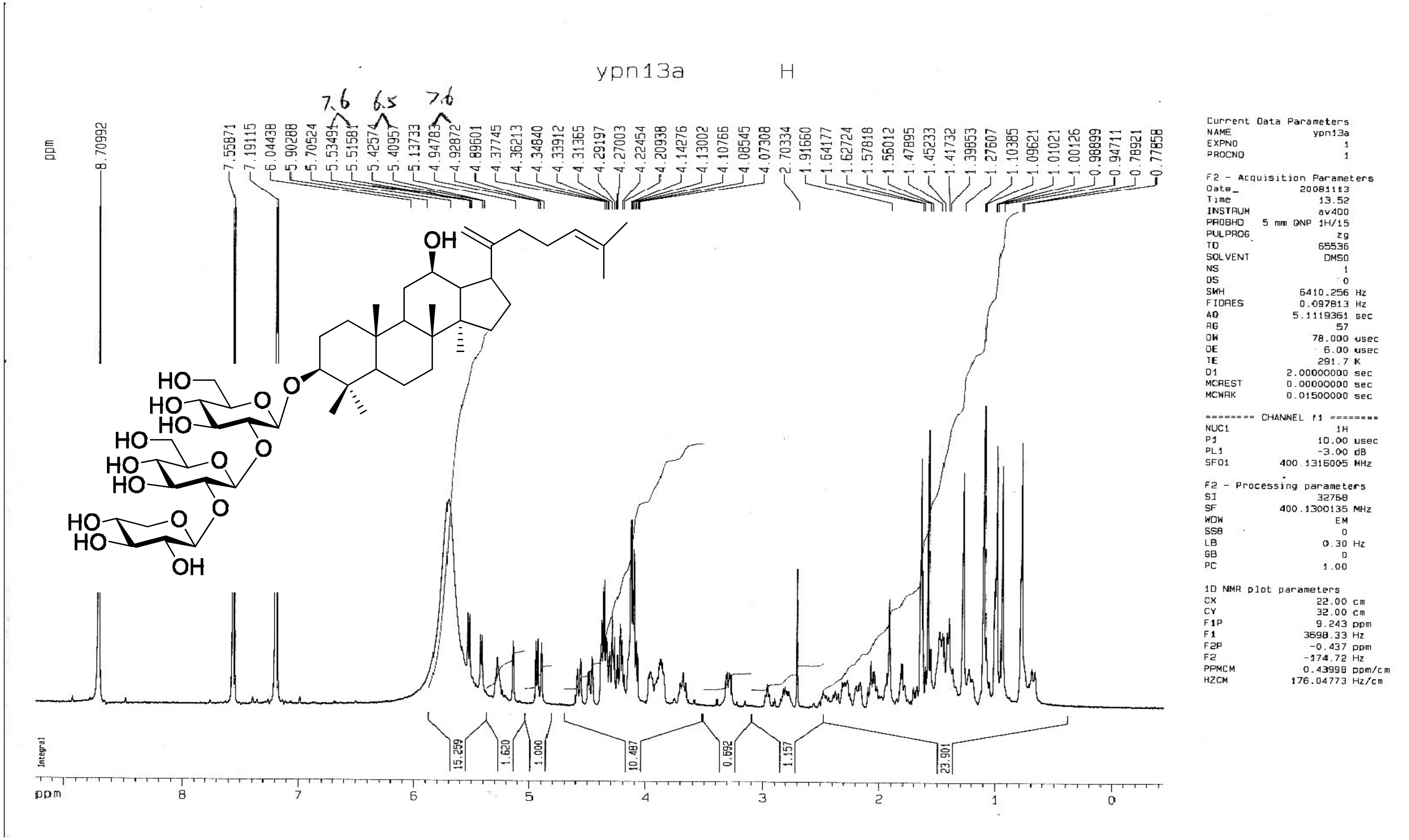

S13 ^{13}C NMR spectrum for notoginsenosides SFt$_3$ (3) in C_5D_5N

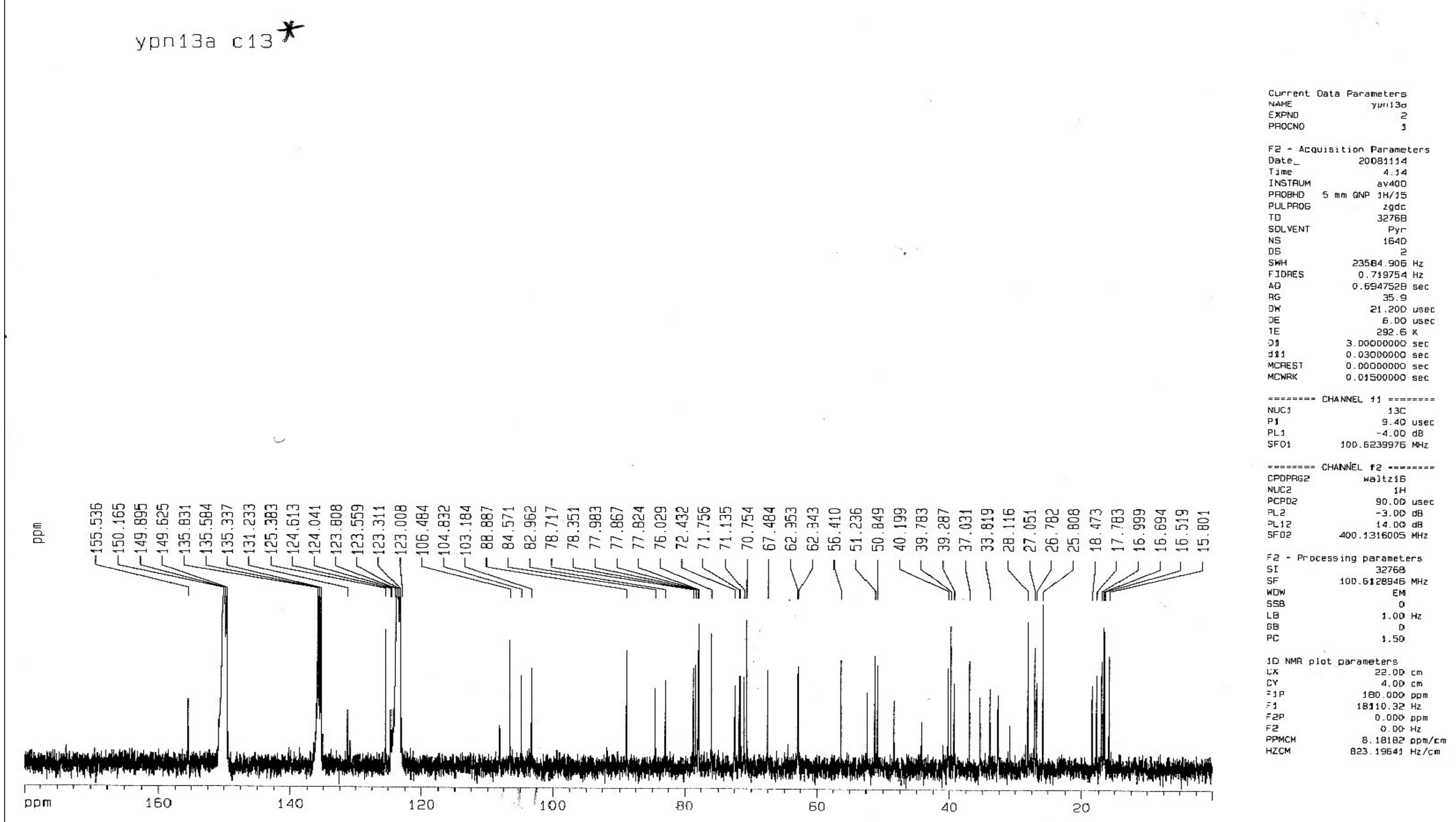

S14 ^{1}H-^{1}H COSY spectrum of notoginsenosides SFt$_3$ (3) in C_5D_5N

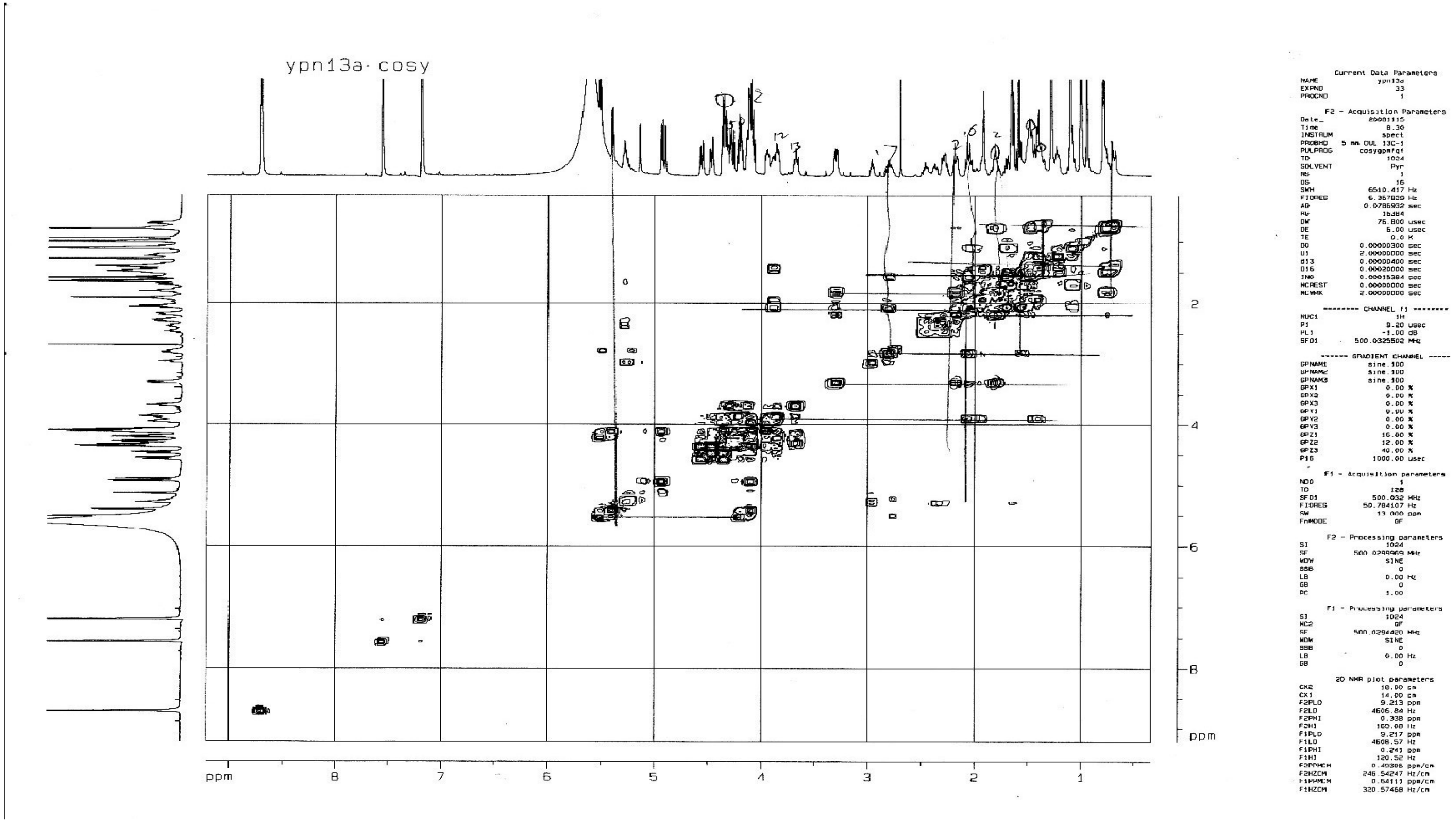

S15 HMBC spectrum for notoginsenosides SFt_3 (3) in C_5D_5N

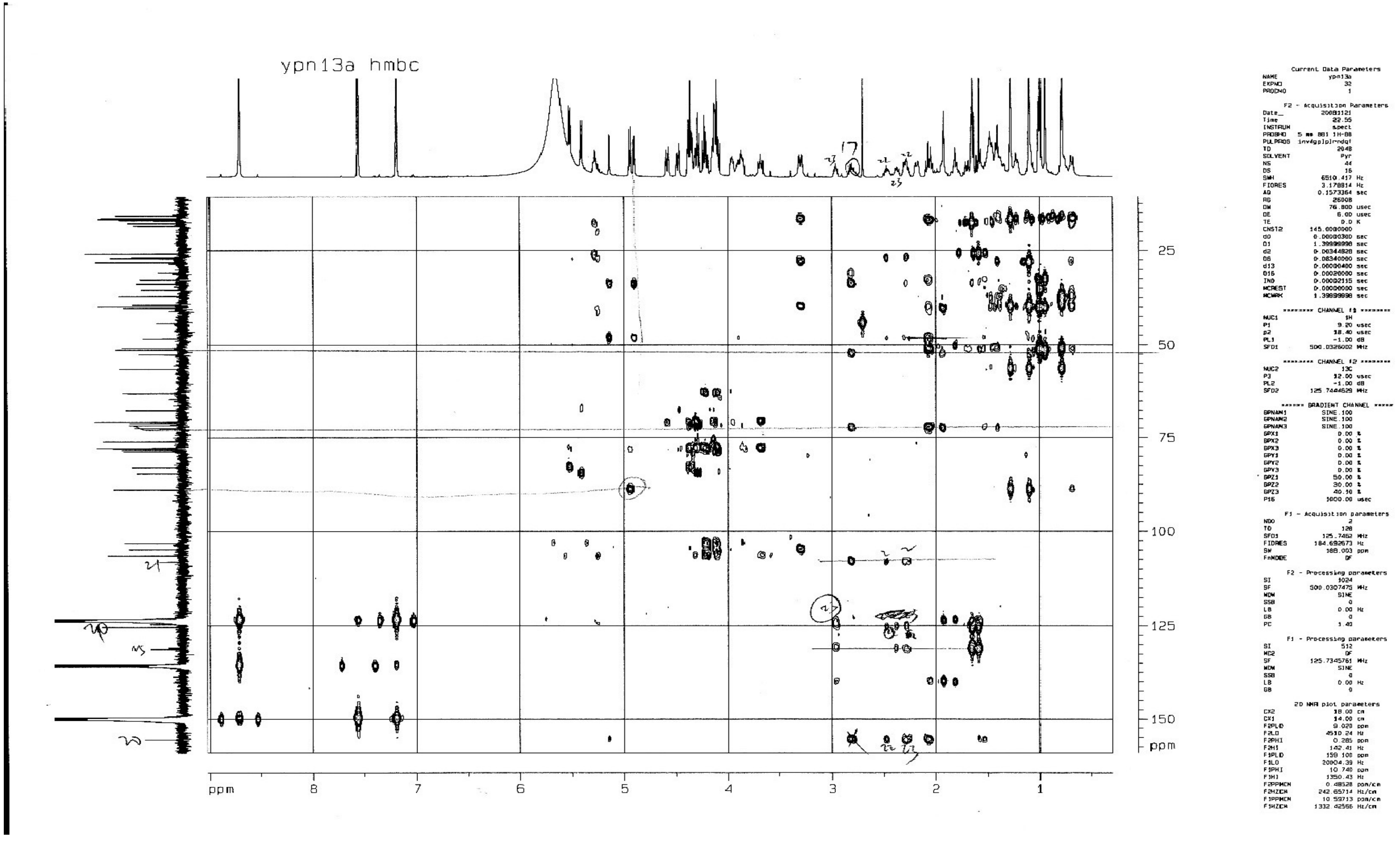

S16 ^{1}H NMR spectrum of notoginsenosides SFt$_4$ (4) in C_5D_5N

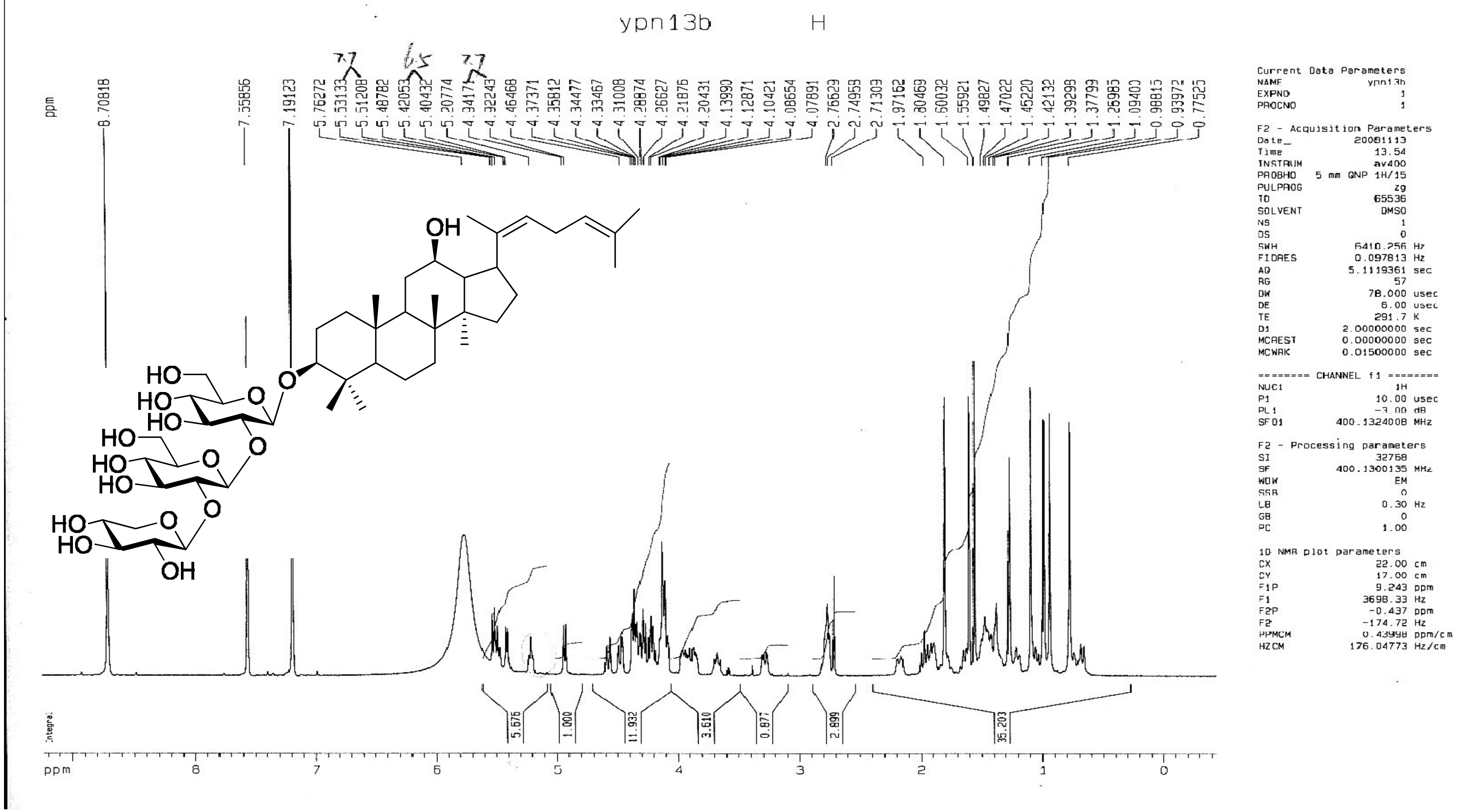

S17 ^{13}C NMR spectrum for notoginsenosides SFt$_4$ (4) in C_5D_5N

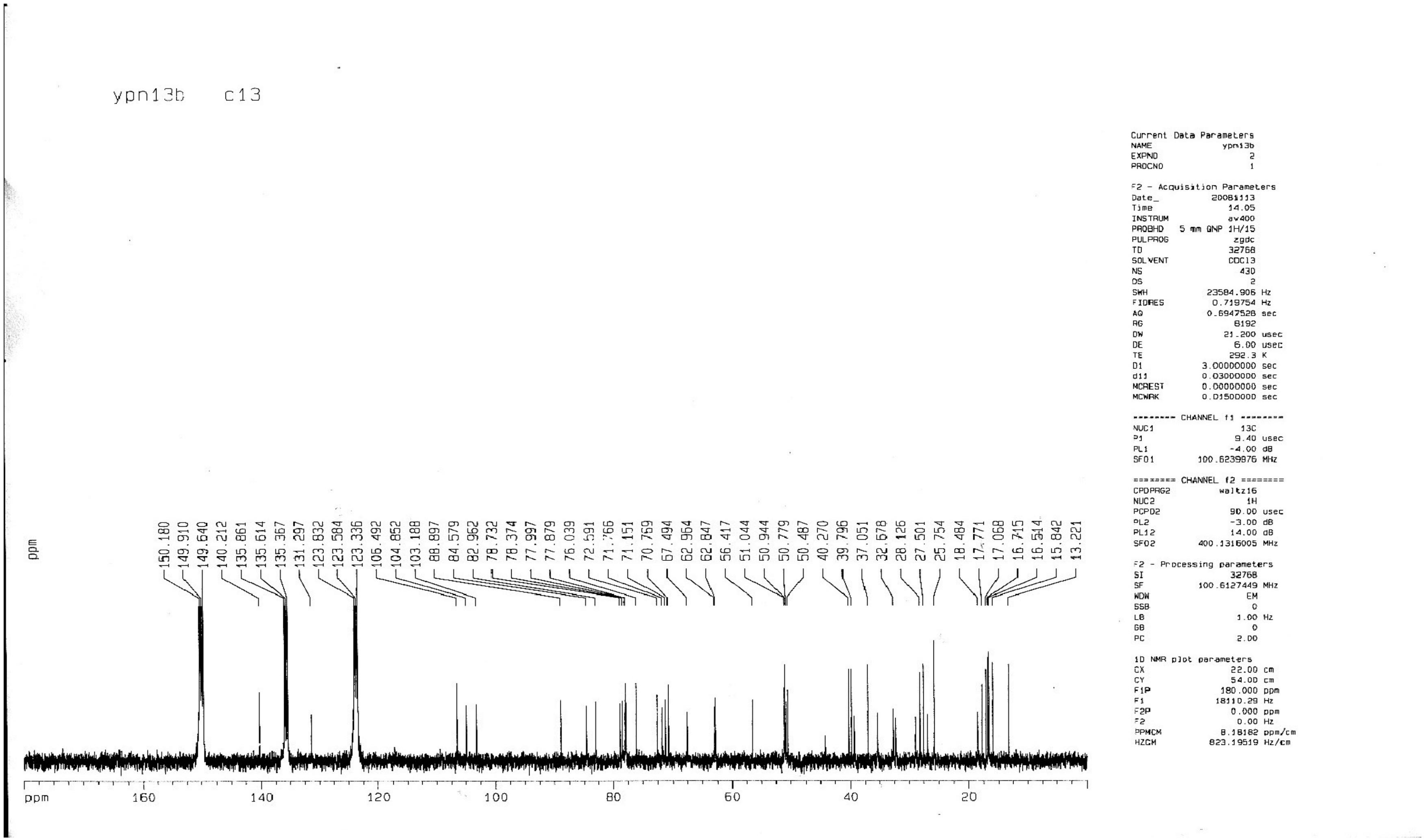

S18 ^{1}H-^{1}H COSY spectrum of notoginsenosides SFt_4 (4) in C_5D_5N

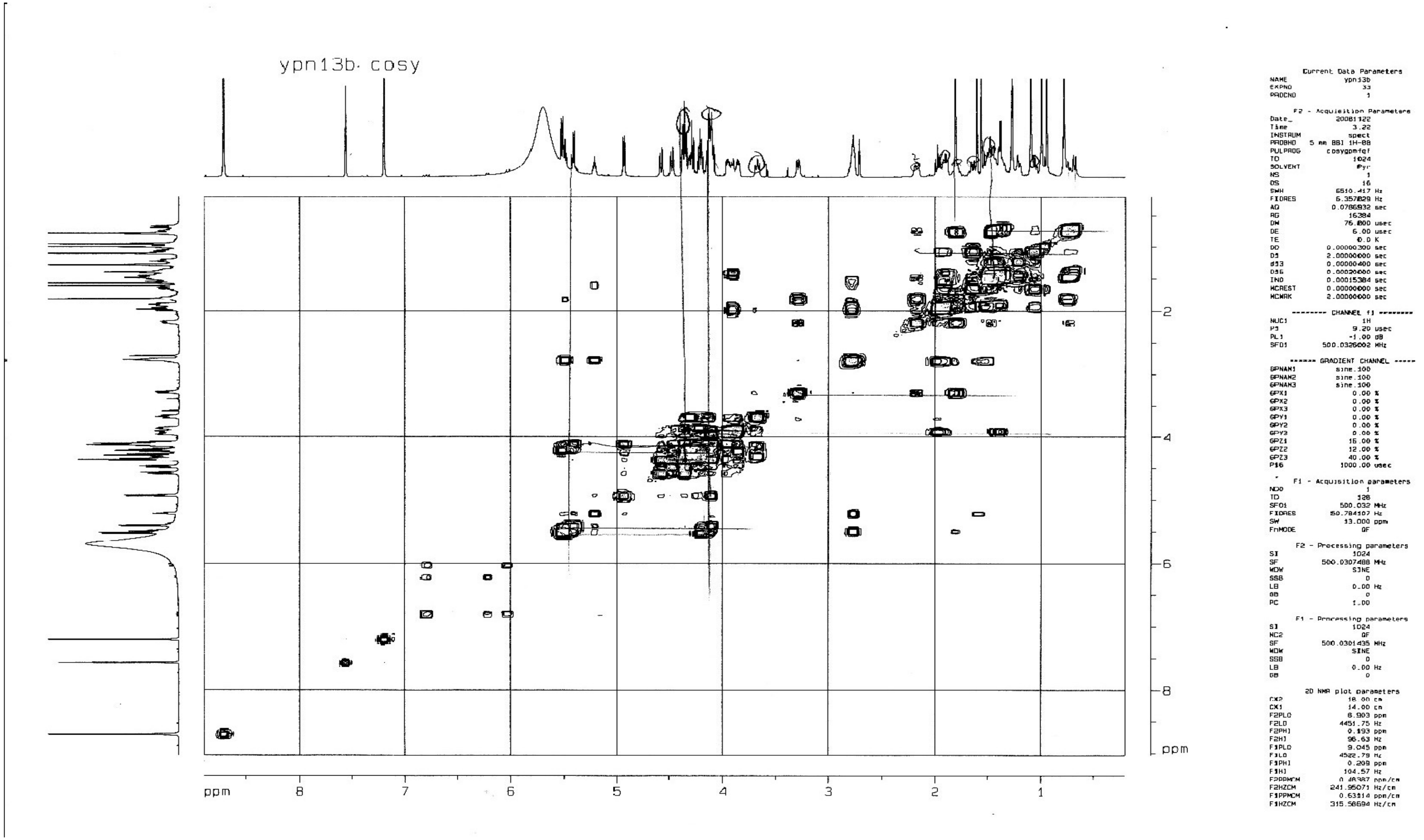

S19 HMBC spectrum for notoginsenosides SFt$_4$ (4) in C_5D_5N

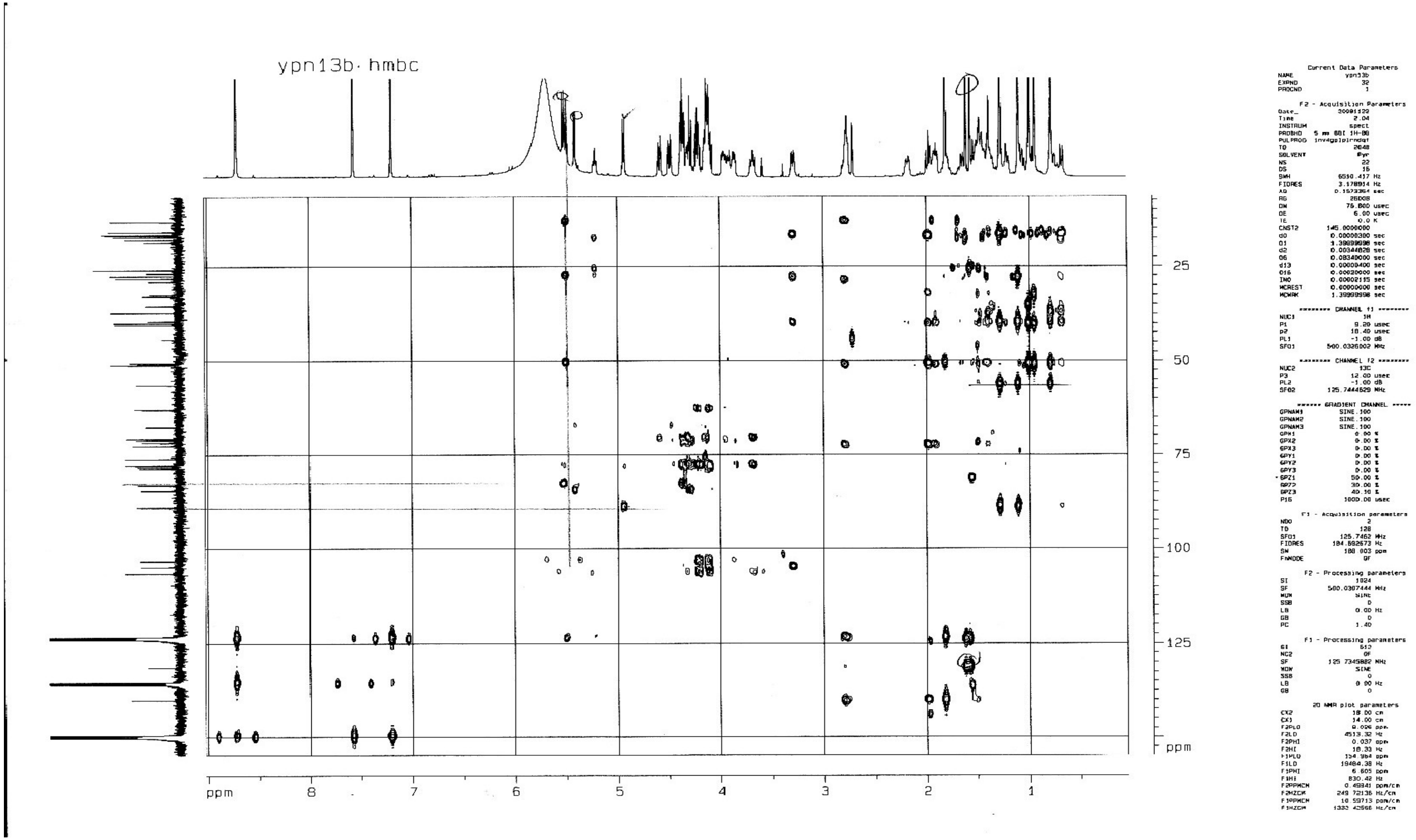

Supplement: Supplementary file 1 — Supplementary material, approximately 4.43 MB. [file 13659_2011_36_MOESM1_ESM.pdf]
